# Tumor Microenvironment: Recent Advances in Immunotherapies of Pancreatic Cancer

**DOI:** 10.3390/medicina61101776

**Published:** 2025-10-01

**Authors:** Sharon Varghese Thankachan, Vijayalakshmi Jayaraman, Liza Datta, Soniga Apthi, Binish Fatima Zaman, Raghav Gurunathan, Anuppama Suresh, Parthasarathy Chandrakesan, Ramachandran Vinayagam, Sang Gu Kang, Kanagaraj Palaniyandi, Dhanavathy Gnanasampanthapandian

**Affiliations:** 1 Cancer Science Laboratory, Department of Biotechnology, School of Bioengineering, SRM Institute of Science and Technology, Kattankulathur 603203, Indiamuthammal.kanagaraj@gmail.com (K.P.); 2 Department of Medicine, University of Oklahoma Health Sciences Center, Oklahoma City, OK 73104, USA; 3 Department of Biotechnology, College of Life and Applied Sciences, Yeungnam University, 280 Daehak-Ro, Gyeongsan 38541, Republic of Korea; rambio@ynu.ac.kr

**Keywords:** immunotherapy, tumor microenvironment, T cells, B cells, cytokines, immunosuppression

## Abstract

The progression of pancreatic cancer (PC) is significantly influenced by the immune system. In the United States, PC is the third leading cause of cancer-related mortality. The high lethality of PC is attributed to its immunological advantage, which is facilitated by an immunosuppressive microenvironment, a low mutational burden, and minimal T-cell infiltration. Although immunotherapies, such as checkpoint blockades or genetically engineered T cells, have not yet demonstrated viability, there is a growing body of evidence suggesting that innovative combinations of conventional therapies and various procedures may lead to effective immunotherapy in the treatment of PC. This review focuses on the importance of the tumor microenvironment and the promising role of immunotherapies in PC.

## 1. Introduction

Pancreatic cancer (PC) is recognized as one of the most aggressive malignancies. The survival rate of PC is <5 years, with patients exhibiting a survival rate of only 8.2%. By 2030, PC is projected to become the second leading cause of cancer-related deaths. Diagnosed PC is higher in males (53%) than females (47%) [1]. In 2017, PC was the third most common cause of cancer-related mortality in the United States. Regrettably, ~80–85% of PC cases are unresectable. Despite significant advancements in PC research, the prognosis for this disease remains poor. PC demonstrates a high level of resistance to chemotherapy, radiotherapy, and immunotherapy. The tumor microenvironment (TME) of PC is characterized by a dense accumulation of immune cells that suppresses the host immune system. The TME significantly influences cancer progression, metastatic potential, and resistance to therapeutic interventions, which is a concerning aspect of PC. The microenvironment not only contributes to initial resistance to treatments but also to later acquired resistance, extending beyond traditional cytotoxic chemotherapies to include targeted therapies and immunomodulatory interventions (6). Furthermore, the TME’s dense stroma impedes immune cell infiltration and drug penetration [2]. Although chemotherapy has been previously employed, progress has been limited [3]. Consequently, immunotherapy has emerged as the fourth pillar of PC treatment [4]. The exploitation of the host immune system or the introduction of modified immune components into the host a potentially effective therapeutic approach for PC treatment with minimal or no side effects [5]. Currently, several strategies are employed to target the PC immune system: the use of monoclonal antibodies (mAbs) against the tumor antigens, targeting immune components [e.g., tumor-associated macrophages, tumor-associated neutrophils, pancreatic stellate cells (PSCs), tumor-infiltrating leukocytes, and cancer-associated fibroblasts (CAFs)], as well as blocking immune checkpoints, such as programmed cell death protein -1 (PD-1), programmed death-ligand 1 (PD-L1) and CD40 [6,7]. Rather than relying on a single agent, various immunotherapy approaches are utilized to combat PC. For instance, the combination of immunotherapy with chemotherapeutic agents has shown promising results, with most of these combination therapies currently in phase I and phase II clinical trials. Immunotherapy is regarded as more efficacious when combined with chemotherapy rather than with radiotherapy [8]. Although trials of immunotherapies have commenced recently, their effectiveness against PC is constrained by the TME. Adverse effects have been observed, attributed to the inflammation induced by immunotherapeutic agents on the host and the pre-existing immune cell population [9,10].

The immune system plays a crucial role in safeguarding the body against various pathogens and substances. However, translating fundamental immunological knowledge from animal models to humans has proven to be a significant challenge, often resulting in more setbacks than successes [11]. To enhance the understanding of the human immune system, immunologists are diligently employing diverse methodologies and developing novel therapeutic strategies. The fundamental components of the immune system comprise various immune cell types and cytokines, which facilitate intercellular communication. Fortunately, the majority of these components can be accessed using current technological advancements, and a representation of these elements is obtainable from a blood sample, which is readily accessible to researchers.

Over the past decade [12], various immunotherapeutic agents have been employed in the clinical management of tumors. These agents include diverse immune checkpoint blockers (ICBs) that target cytotoxic T lymphocyte–associated protein 4 (CTLA4), PD-1, or its primary ligand PD-L1. They are analogous to homologous T cells engineered to interact with CD19 through the targeting the chimeric antigen receptor (CAR) [13]. Ongoing clinical trials are investigating additional immunotherapies that demonstrate anticancer efficacy. These encompass numerous immunostimulatory mAbs and molecular fragments that counteract cancer-induced immunosuppression, similar to therapeutic vaccines targeting various cancers [14].

Stage I PC patients exhibit a survival rate of ~29%, whereas the overall 5-year survival rate remains at a mere 8% [15]. Relapses are inevitable and the therapeutic options for PC are limited, encompassing radiation, chemotherapy, and surgical interventions. Furthermore, a significant mortality rate is attributed to the delayed diagnosis of the disease. Recent advancements in immunotherapy have transformed cancer treatment paradigms, particularly for melanoma [16]. The interaction between the immune system and cancer cells occurs through the stages of elimination, equilibrium, and escape. During the elimination phase, the immune system identifies and eradicates altered cells [17]. As transformed cells transition from the elimination phase to the equilibrium phase, they undergo genomic alterations and establish a TME conducive to the development of early lesions. In the escape phase, cancer cells select immunosuppressive cells, including myeloid-derived suppressor cells (MDSCs), regulatory CD4^+^FOXP3^+^ T cells (Treg cells), and cancer-associated macrophages [18]. This review incorporates the latest research on various immunotherapies and examines the impact of TME on PC.

## 2. Tumor Microenvironment

**Figure 1:**
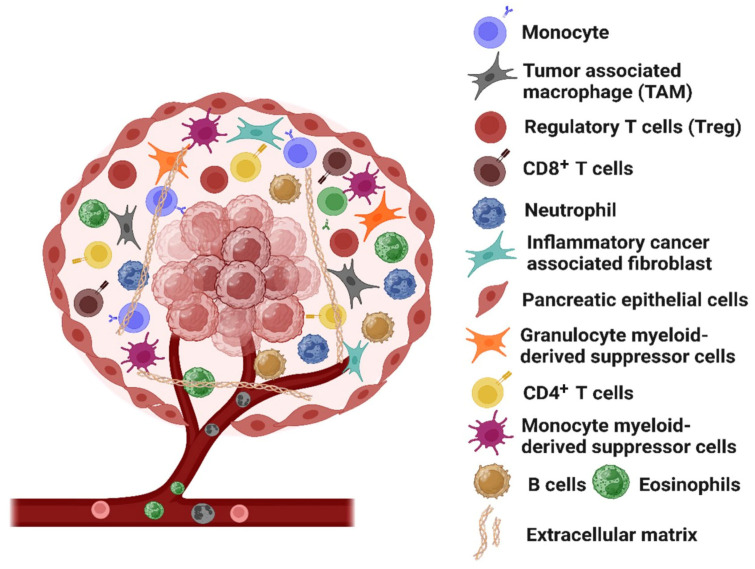
The tumor microenvironment (TME) in pancreatic cancer (PC): The TME in PC plays a critical role in tumor progression through various mechanisms. The desmoplastic stroma, comprising cancer-associated fibroblasts and the extracellular matrix, facilitates cancer growth and progression both directly and indirectly. Additionally, immunosuppressor cells, such as Treg and tumor-associates macrophages (TAM), inhibit CD8^+^ T cells, which are pivotal in the anti-tumor immune response, thereby creating immunosuppressive TME.

The enhanced understanding of the critical characteristics of the TME in eliciting immune responses against cancer has significantly advanced the field of immuno-oncology [19,20]. The concept of “immune context” has been introduced and validated in light of these findings, leading to the classification of tumors into four categories: cold, immunosuppressed, excluded, and heated [21,22]. It has been suggested that “hot” tumors may respond effectively to immune checkpoint inhibitors (ICIs) if they possess growth-promoting lymphocytes, genomic susceptibility, and a prior antitumor immune response, as indicated by complementary determinations such as the expression of anti-PD-L1 on cancer-associated immune cells [23]. Cold tumors do not easily provoke an immune response and are frequently resistant to treatment [24]. Conversely, “cold” tumors have been identified as immunologically “ignorant,” characterized by insufficient invasion, increased proliferation, minimal expression of antigen presentation machinery, including major histocompatibility complex I (MHC I), and limited mutational perturbations (minimal expression of neoantigens) [25]. The transition from “cold” tumors to generative “hot” cancers that are susceptible to ICIs is a dynamic area of research. In this context, targeting the interaction between cancer and T cells may enhance resistance in an inhospitable microenvironment and contribute to the efficacy of immunotherapies [26]. Radiation and chemotherapy have been employed to potentially convert “cold” tumors into “hot” ones by increasing the antigenicity and priming ability of malignancies. Ionizing radiation-induced antigen release and immunogenic cell death may transform tumor cells into an in situ vaccine [27]. There is evidence that ICIs directly influence the metabolic landscape in the TME, thereby affecting the function of effector T cells (Figure 1). Increasing evidence suggests that the TME sustains inappropriate metabolic reprogramming, which impairs T-cell activity and diminishes the immunological response to tumors [28,29].

PC is characterized by a well-documented stromal response, which encompasses a diverse array of cells, including PSCs, immune cells, and other components that secrete growth factors [30]. Key stromal cell types implicated in cancer progression include PSCs, CAFs, regulatory T cells, and MDSCs. The mononuclear phagocyte within the bone marrow is capable of generating macrophages, a crucial subset of innate immune cells that contribute to PC immunosuppression and desmoplasia. Environmental signals, such as inflammatory cytokines like IL-8, IL-6, IL-1β, and IL-10, may activate TAMs [31]. Furthermore, a high infiltration of TAMs has been associated with tumor progression, prognosis, and patient survival in PC [32].

Metastasis is the primary factor contributing to the high mortality rate among patients with PC, with mechanisms of metastasis including hematogenous spread, local invasion, and lymphatic dissemination. The majority of patients diagnosed with pancreatic malignancy for the first time present with locally advanced and unresectable stage. It has been documented that most patients succumb to liver, lung, or peritoneal metastases [33]. The immune cells within the body, such as natural killer (NK) cells and CD8^+^ T cells, have the capacity to counteract or even eliminate malignant cells during metastasis, even at the early stage of epithelial–mesenchymal transition (EMT) [34]. Nevertheless, PC cells can evade immune surveillance and metastasize to distant sites due to the modulation of immunosuppressive components within the TME [35]. Although previous research has focused on the intrinsic properties of cancer cells, recent clinical investigations have identified the cancer stroma within the TME as playing a significant role in the pathogenesis of PC, potentially maintaining the malignant phenotype and promoting metastasis. Numerous studies have explored the mechanisms of PC metastasis, and recent findings indicate that key particles and mechanisms within the immunologic microenvironment are closely associated with pancreatic disease metastasis [36].

TME is crucial in the emergence of resistance to various treatments, including immune checkpoint blockage therapies (ICT) [37]. Patients receiving immune checkpoint inhibitor (ICIs) frequently show signs of immunological memory commonly [38]. Although initial ICIs treatments can yield improved outcomes in the early stages, resistance often arises when ICIs are used alone [39]. A variety of resistance mechanisms have been discovered [40].

### 2.1. The Role of Pancreatic Cancer TME in Metastasis

PC-TME is distinguished by a structure that includes PSCs, which are vital in metastasis, budding tumor cells, and a partial EMT state [41]. Tumor budding is indicative of a partial EMT state, as evidenced by features such as reduced expression of E-cadherin, loss of β-catenin expression, and increased expression of EMT markers, including zinc finger E-box-binding homeobox proteins 1/2 (ZEB-1/2), SNAIL, and N-cadherin [42,43]. The absence of both proliferative and apoptotic markers further confirms the presence of an EMT subtype, suggesting that proliferation is incompatible with EMT. Moreover, tumor budding has been linked to the dysregulation of the A-200 family miRNA, which is involved in tumor suppression mechanisms related to cell transformation, invasion, migration, and tumor growth and metastasis [44]. Hypoxia-inducible factor (HIF-1) regulates ATP utilization, reactive oxygen species (ROS) formation, and glucose redirection for energy production, thereby metabolically reprogramming the TME to facilitate disease progression [45]. TAMs influence metastasis by secreting macrophage-induced protein-3α (MIP-3α), which modifies the extracellular matrix (ECM) structure [35]. Hypoxia is also associated with the overexpression of heme oxygenase-1 (HO-1) in PC [46]. Recent findings suggest that inhibiting HO-1 may enhance the efficacy of chemotherapeutic agents such as gemcitabine and paclitaxel [47]. Furthermore, the development of an immunosuppressive TME and the lack of interaction between immune cells and pancreatic liver metastatic cells have been observed, promoting liver metastasis [48]. This phenomenon supports the establishment of a pro-TME during the metastatic process, involving CAFs RGS5^+^, neutrophils S100A8^+^, FOXP3^+^ regulatory T cells, and CCL18^+^ lipid-associated macrophages [48].

### 2.2. Lymphatic Metastasis

Metastatic hematopoiesis is frequently observed in PC, akin to other solid tumors. Acute surgical patients often exhibit inherent peripancreatic invasion, particularly into the post-pancreatic and extra-pancreatic networks. A recent study identifies lymph node metastasis as a predictive factor for pancreatic ductal adenocarcinoma (PDAC) [49]. Research by McKay et al. demonstrates that lymphatic metastasis significantly impacts survival following complete resection of pancreatic head cancer (PHC). Patients without lymphatic metastases show markedly higher 5-year survival rates compared to those with such metastases [50]. The invasion of lymphatics and subsequent transition to lymph nodes are critical events in the progression of PC. Although lymphatic invasion and lymph node metastasis do not directly increase morbidity in patients with PDAC, they are significant indicators of the disease’s metastatic potential [51]. Furthermore, PC cells derived from patients exhibit an enhanced ability to metastasize to lymph nodes in mouse models [52]. In clinical practice, lymph node status is utilized to predict survival, determine optimal treatment strategies, and monitor disease progression. PDAC carcinomas are often characterized by hypovascularity, with only a few lymphatic and blood vessels interspersed among the tumor cells [53].

### 2.3. Biomarkers for TME

The most prevalent dense structures within the TME are CAFs. CAFs typically constitute all fibrotic tissues that form the TME [54]. These fibroblasts primarily originate from pre-existing PSCs and represent potential therapeutic targets in PC treatment. The presence of glial fibrillary acidic protein (GFAP), desmin, vitamin A lipid droplets, and acetylcholine receptors signifies the existence of PSCs [55]. Given the intrinsic heterogeneity of CAFs, biomarkers are not exclusive to a single cell subtype. Nevertheless, specific biomarkers have been identified for CAF detection, including α-SMA, vimentin, fibroblast activation protein (FAP), platelet-derived growth factor receptor α/β (PDGFR α/β), and podoplanin (PDPN/gp38) [56,57]. Extracellular signal-regulated kinases (ERK) are highly expressed in PSCs, facilitating metastasis and interaction with the cancer stroma within the PC milieu [58]. Inhibition of ERK1/2 specifically results in diminished EMT processes, increased markers of cellular senescence, and activation of autophagy in PSCs of PC [58]. Leukemia inhibitory factor (LIF) serves as a more effective biomarker compared to carcinogenic embryonic antigen (CEA) and carbohydrate antigen 19-9 (CA 199), with case studies indicating high LIF expression in patients with lymph node metastases, correlating with decreased overall survival and recurrence-free survival [58,59]. Elevated expression of CD10^+^ PSCs is indicative of lymph node metastasis. Furthermore, vascular endothelial growth factor (VEGF) subtypes C/D are crucial for lymphatic vessel formation and thus represent viable targets [35]. Recently, kinesin family member 5B (KIF5B) and secreted frizzled-related protein (SFRP2) have been identified as components of the TME in PC [60].

## 3. Characteristics of TME in Pancreatic Cancer

PDAC is characterized by its aggressive nature. A prominent feature of TME is the dense stroma, which imparts both an aggressive phenotype and immunosuppressive properties. This is influenced by several factors, including CAFs, PSCs, and the hypoxic environment [61,62]. Smooth muscle antigen alpha (SMA-α) and FAP are integral to the wound healing process within the ECM [63]. In addition, the TME comprises predominant cell types, such as certain macrophages, cytotoxic immune cells, regulatory cells, endothelial cells, and PC-specific neuronal cells [64]. The ECM, rich in collagen, proteoglycans, hyaluronic acid, and fibronectin, is abundant within the TME [65]. It also serves as a source of hepatocyte growth factor (HGF) and fibroblast growth factor (FGF) [66].

### 3.1. Pancreatic Stellate Cells

PSCs represent a subset of cells integral to the dense TME of the PC [67]. These stellate cells, which store vitamins, are located around the periductal regions of the pancreas. In their quiescent stage, PSCs play a role in regulating endocrine and exocrine secretions, facilitating phagocytosis, and maintaining normal pancreatic morphology [68]. Upon activation, PSCs exhibit increased expression of ECM proteins, including laminin, collagen, and fibronectin, which contribute to the fibrotic nature of pancreatic tumor tissue. Furthermore, activated PSCs (aPSCs) facilitate the recruitment of immunosuppressive cells and are implicated in the progression of non-invasive pancreatic intraepithelial neoplasms to invasive PDAC subtypes [69]. The transition from PSC to aPSC is influenced by lifestyle factors (smoking and alcohol consumption), environmental stressors (hypoxia, hypoperfusion, and oxidative stress), which lead to the secretion of cellular factors (IL-1, IL-6, HIF-1α, TGF-β, and CTGF), and specific molecular signals, including Wnt/β-catenin and PI3 pathways [68]. In addition, aPSCs contribute to the recruitment of immunosuppressive cells through the secretion of IL-6 and macrophage colony-stimulating factor (M-CSF) [70]. The expression of autophagy factors is essential for aPSCs, and the presence of the autophagosomal marker microtubule-associated protein-1 (MAP1) in PSCs has been associated with poor patient survival rates [71].

### 3.2. Cancer-Associated Fibroblasts

CAFs are integral components responsible for the dense desmoplasia characteristic of the TME, contributing to its aggressiveness [44]. These CAFs originate from various mesenchymal cell types, with PSCs serving as the primary source [72]. Predominantly, resident PSCs differentiate into CAFs. The transition of CAFs towards cancer is marked by a reduction in FAPs, the expression of α-SMA, and the acquisition of a contractile morphology [73]. In PCs, CAFs exhibit significant heterogeneity, comprising multiple subtypes derived from PSCs that secrete cytokines IL-6 and α-SMA at low levels [56]. This heterogeneity poses a challenge for therapeutic targeting, as it can lead to epigenetic regulation, induction of stem cells in PC, and metabolic reprogramming [57]. Recent investigations employing single-cell RNA sequencing have elucidated the heterogeneity of stromal components within the TME, with CAFs playing a pivotal role in TME regulation. Cancer-derived substances, such as interleukin-1 (IL-1) and transforming growth factor-beta (TGF-β), have been demonstrated to alter the phenotype of adjacent fibroblasts, inducing their differentiation into inflammatory and myofibroblastic CAFs, respectively. Inflammatory CAFs secrete IL-6 and facilitate tumor growth, whereas myofibroblastic CAFs contribute to the formation of the surrounding stroma. These distinct stromal cell subtypes correlate with specific cancer cell subtypes, reflecting the capacity of cancer cells to shape a microenvironment conducive to their survival [74].

### 3.3. Tumor-Associated Macrophages

Macrophages, which primarily differentiate from monocytes produced in the central bone marrow, are present in both healthy and inflamed tissues [75]. The metabolism of macrophages plays a crucial role in regulating the progression of PC. The promotion of glycolysis by HIF-1 leads to increased expression of IL-1. To enhance phagocytosis, M1 macrophages exhibit modifications in the tricarboxylic acid (TCA) cycle, increased glycolysis, and oxidative phosphorylation (OXPHOS). Phagocytosis and inflammation are more effectively facilitated by lipid metabolism. The progression of PDAC is affected by impaired glutamine metabolism. These findings underscore the significance of macrophage metabolism in PC [76]. Certain macrophages are specialized, such as Kupffer cells in the liver and splenocytes in the white pulp of the spleen. Circulating macrophages are also present in the TME, aided by various molecules, including chemokines and cytokines, along with high concentrations of lactic acid, local anoxia, and environmental factors [77,78]. Collectively, these cells constitute TAMs. TAMs are classified into M1 and M2 types based on their polarizing properties. Although M1 macrophages are involved in the secretion of pro-inflammatory cytokines, M2 macrophages are associated with the secretion of anti-inflammatory cytokines [79]. A previous study on the blockade of CCR4^+^ in TAMs demonstrated that TAMs are essential for the infiltration of immune cells into the tumor [80]. An alternate study of examining organ-specific isotypes of PDAC revealed a significant increase in Maf+ macrophages, which is directly associated with CD8^+^ T cells, thereby promoting an immunosuppressive microenvironment [81].

### 3.4. Hypoxia

In contrast to other solid tumors, PC is characterized by a substantial presence of stromal cells and an ECM, yet lacks vascularization, resulting in severe and persistent hypoxia within the tumor. This hypoxic microenvironment significantly influences the biological characteristics of PC, including metabolic reprogramming, cancer stem cell dynamics, invasion and metastasis, and pathological angiogenesis, all of which collectively contribute to disease progression and therapeutic resistance [62]. Consequently, hypoxia is a critical aspect of the pancreatic TME due to the dense stroma that impedes the infiltration of immune cells and other therapeutic agents [45]. A recent study employing a xenograft model demonstrated that under hypoxic conditions, HIF-1α induces LncRNA-BX111, which subsequently leads to the transcription of ZEB-1, an EMT inducer [82]. Inhibition of BX111 results in the suppression of tumor growth and metastasis [82]. In addition, the hypoxic environment facilitates pH balance and glycolysis regulation by enhancing the expression of activated KRAS, which in turn leads to the overexpression of carbonic anhydrase 9 (CA9) through modulation of HIF-1α and HIF-2α [83]. Recent studies have shown that specific molecular and cellular alterations occur in PC under hypoxic conditions. Additionally, hypoxia downregulates lncRNA-CF129, which plays a pivotal role in preventing invasion in a FOXC2-dependent manner via the ubiquitination and degradation of p53 [84,85,86,87]. CF129 is transcriptionally downregulated by hypoxic factors, such as the HIF-1α/ histone deacetylase 1 (HDAC1) complex, thereby contributing to disease progression [88]. Activation of the phosphatidylinositol-3-kinase (PI3K)-mTOR pathway under hypoxic conditions inhibits NK cells from eliminating tumor cells. Moreover, by upregulating the expression of matrix metalloproteinase10 (MMP10), hypoxia diminishes the expression of the tumor cell surface recognition molecule MICA, which downregulates the expression of NK and group 2D NK cells (NKG2D) on T cells, facilitating immune evasion from tumor cells [89,90]. Various cellular components contribute to the distinct properties of the TME. In addition, increased production of vascular endothelial growth factor (VEGF) under hypoxic conditions promotes angiogenesis by inducing endothelial cell proliferation and the formation of new blood vessels [91,92,93,94].

### 3.5. Lymphangiogenesis and Lymphatic Metastasis

One of the most critical components of PC metastasis is the TME. The disease disseminates either through local metastasis or lymph node metastasis [95]. Lymphatic metastasis, facilitated by lymphangiogenesis, represents a successful mechanism by which cancer metastasizes to both distant and local areas. A high density of immune cells, including regulatory T cells (Tregs), immature and tolerogenic dendritic cells (DCs), cytokine producers, and MDSCs, in the lymph nodes at tumor drainage sites, is associated with lymph node metastasis. M2-type TAMs have been identified as playing a crucial role in the regulation of lymphangiogenesis [96]. Furthermore, PSCs and PC cells initiate angiogenesis by secreting pro-angiogenic factors, leading to the formation of microcapillaries [93,97,98,99]. Extracellular vesicles (EVs) have been extensively reported to promote cancer progression and structural alterations in the TME, as well as metastasis to adjacent organs [100].

### 3.6. Functional Diversity of Tumor Lymphangiogenesis

The extent to which lymphangiogenesis contributes to the TME remains uncertain, despite its role in promoting tumor dissemination in both primary tumors and empty lymph nodes (LNs). Recent research employing phylogenetic reconstruction techniques has revealed that the majority of colorectal cancer metastases to distant organs bypass the LN [101]. Historically, lymphatic metastasis was considered a passive process. However, emerging evidence indicates that lymphatic endothelial cells (LECs) actively participate in the invasion of cancer cells into lymphatic vessels (LVs) and their subsequent infiltration into LNs [51]. One study showed that LEC extensively form filopodia in response to VEGF-C, which facilitates the shredding of tumor cells into the lymphatic vasculature [102]. Notably, LECs promote the migration of tumor cells into the lymphatics, with chemokines playing a contributory role. For instance, CXC-motif chemokine 12 (CXCL12)-expressing LVs have been shown to enhance the invasiveness of various cancer cell types expressing CXCR4. Similarly, the production of secondary lymphoid-tissue chemokine (CCL21) by LECs has been implicated in the relocation of CCR7+ malignant cells to a niche within the LV during tumor progression [103,104,105,106]. In PDAC, CD3^+^ markers (90%) are indicative of tumor-infiltrating lymphocytes (TILs) [88], predominantly of the CD4^+^ subtype. Most TILs are CD45RO^+^ and CCR7^−^, suggesting that these TILs are effector cells with known antigenic determinants. In addition to TILs, CD8^+^ cells express CD28 and, to a lesser extent, Glucocorticoid-induced TNFR-related protein (GITR), a receptor that supports CD8^+^ proliferation [107]. TILs in PC can enhance therapeutic responses by recognizing and capturing tumor-associated antigens [108]. While TILs may also include B cells, these typically remain inactive within the tumor [109].

### 3.7. T Lymphocytes

T cells are systematically categorized based on their T cell receptor (TCR) subunits, similar to central lineage markers. T cells within the classification are capable of recognizing proteins presented on the cell surface via MHC I or II (CD8 or CD4 T cells, respectively) due to the αβ TCR complex [110]. To date, the majority of research has concentrated on CD4^low^ CD8^high^ T cells, a subset believed to originate from peripheral CD8 T cells that co-express low levels of CD4 following activation [111].

Owing to their well-documented antiviral and anticancer properties, CD8^+^ T cells are also referred to as cytotoxic T lymphocytes (CTLs). CTLs are capable of releasing substantial quantities of antitumor cytokines and cytotoxic particles, such as TNFα and granzymes [110]. The memory T cell subsets also exhibit a range of divisions, from cells with a more naïve phenotype to those with an effector-like phenotype. These cells typically align with the lineage of effector memory T (T_EM_), stem cell-like memory T, and effector memory RA^+^ T cells [112]. Tissue-resident memory T cells (T_RM_) in the surrounding tissues interact with canonical markers such as CD103, also called integrin αE, CXCR6, CD49a, and CD69 [113,114,115].

### 3.8. B Lymphocytes

In the realm of clinical immunotherapy, patient stratification and response evaluation have predominantly concentrated on T-cell responses. Recent research, however, has recognized the significance of B lymphocytes in immunotherapy, linking their quality to improved prognoses in various cancers, including melanoma, breast cancer, renal cell carcinoma, colorectal cancer, hepatocellular carcinoma, and squamous cell carcinoma of the head and neck [116,117,118,119]. Within the TME, B lymphocytes can exhibit either pro-tumor or antitumor characteristics [100]. In ectopic lymph nodes, such as tertiary lymphoid structures (TLS), tumor-infiltrating B cells differentiate into memory B cells and IgG1-producing plasma cells with the assistance of follicular Tfh cells. The IgG1 and IgE antibodies produced by memory and plasma B cells in response to tumor-associated antigens activate the complement system, phagocytosis, or antibody-dependent cytotoxicity of NK cells or macrophages [120,121]. A defining feature of proficient antigen-presenting cells (APCs) is their ability to internalize antigens and present the processed antigen on MHC class molecules to T lymphocytes [110]. Although B cells possess the capability to function as APCs, their efficacy appears inferior to that of DCs, likely due to their less effective nonspecific antigen uptake. B cells exhibit heightened sensitivity to antigens at lower concentrations compared to DCs, attributed to their typically high-binding affinity (multivalence) upon antigen encounter [122]. Prior to immunization, antigen-specific B cells are exceedingly rare in comparison to DCs, leading to the perception that B cells contribute only modestly to APC-mediated activation of naïve CD4^+^ T cells [123]. Nevertheless, utilizing virus-like particles identified by RNA phage Qβ as a model for nanoparticle antigens, Hong et al. [124] showed that B cells, rather than DCs, are responsible for the initial activation of CD4^+^ T cells and the subsequent differentiation of CD4^+^ T cells into CD4^+^ T follicular helper (TFH) cells. Furthermore, this model can induce a germinal center reaction in the absence of DCs [43].

## 4. Immune System and Immunobiology of Pancreatic Cancer

**Figure 2:**
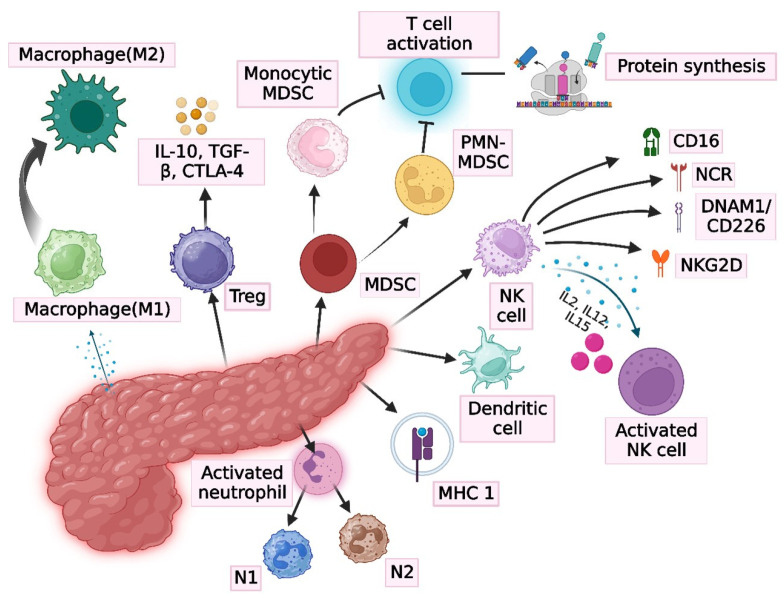
The immune system in pancreatic cancer. Various immune cells, including macrophages (M1 and M2), monocytes, dendritic cells, natural killer cells (NK cells), regulatory T cells (Treg), and neutrophils, modulate PC.

The immune system of PC primarily relies on the components of innate immune cells, including macrophages, myeloid cells, neutrophils, and T lymphocytes. Myeloid cells are present as MDSCs, which exist in two forms: polymorphonuclear MDSCs (PMN-MDSCs), phenotypically similar to neutrophils, and monocytic MDSCs (m-MDSCs), resembling monocytes (Figure 2) [125]. Their primary function is the suppression of the immune system through cysteine detachment mechanisms and the upregulation of arginase expression with concurrent downregulation of L-arginine, both essential for T-cell activation and T-cell protein synthesis, respectively [77]. Macrophages, in the form of TAMs, constitute another significant component of the immune system. The TME of PC induces primary macrophage M1 (pro-inflammatory) to differentiate into M2 (anti-inflammatory) subtypes. Tregs are abundant in the PC immune system and exert a suppressive role by secreting factors such as IL-10 and TGF-β and expressing CTLA-4 [126]. Typically, the immune environment of PC is characterized by reduced numbers of DCs, NK cells, and MHC class 1 molecules, which serve as the initial defense against cancer. The suppressive nature of the tumor results in a diminished presence of these cells [127]. NK cells, as innate immune cells, express CD56^+^ receptors and lack T-cell receptors. They are recognized for targeting virus-infected tumor cells. This anticancer activity of NK cells occurs independently of stimulation but it is facilitated by predominantly present receptors, such as CD16, NKG2D, DNAM1 (DNAX accessory molecule), and NK cell receptors [128]. NK cells can be activated by cytokines such as IL-2, IL-12, and IL-15 [129]. Furthermore, the tumor contains neutrophils, a type of granulocyte. PC can recruit neutrophil granulocytes into the solid tumor, but rather than eliciting an antitumor response, the neutrophils promote disease progression [80]. The neutrophil subtypes N1 and N2, referred to as tumor-associated neutrophils (TANs), are present in the early stages of PC and are known to regulate tumor development through various mechanisms, including proinflammatory cytokine release, angiogenesis, and invasion [77]. In a case study involving 112 patients with PDAC, 25 of whom were long-term survivors, the majority of components were identified as CAFs in a quiescent state. In addition, the long-term survivors exhibited increased levels of CD3^+^, CD4^+^, and iNOS^+^ cells (M1 macrophages and neutrophils) and a decrease in the expression of Tregs, CD68^+^ macrophages, and Foxp3 variants [130].

Several secretory factors such as cytokines (IL-6, IL-10 and IL-11, IL-12, GM-CSF and TNF-α), chemokines CXCL-8, CXCL-10, CXCL12 and CCL-3 and growth factors VEGF and TGF-β are important roles in tumor cell invasion and infiltration [131,132]. KRAS mutated PC caused several downstream signaling molecules to be activated and further inflammatory cytokines IL-6, IL-10 and chemokines CXCL2 were activated [133]. In addition, p53 mutation activates the immune evasion in PC [134].

## 5. Immunotherapy

The characteristic features of PC include an immunosuppressive milieu and a dense stroma, which serves both as a physical barrier to drug penetration and as a dynamic component in the regulation of the immune system. Consequently, the immune system is considered integral to the development of PC [135]. In cancer immunotherapy, specific components are administered to either stimulate the immune system to target a tumor or direct these immune components against the tumor, thereby exerting anticancer effects. The first successful immunotherapy was y carried out in 1981 using streptococcal organisms against bone and soft tissue tumors [136]. This was followed in 1984 by immunotherapy against metastatic melanoma in a 33-year-old woman, who underwent several treatments before receiving a recombinant IL2 (rIL2) infusion. After two months, the tumor size ceased to decrease [137,138]. Immunotherapy has now been established as the fourth pillar in the treatment of PC, alongside surgery, chemotherapy, and radiotherapy [139]. Most PC immunotherapies are currently in phase II trials, with a significant number of phase I trials completed (Table 1) [98,140,141,142,143,144,145,146,147,148,149,150,151,152,153,154,155]. These therapies show promise, particularly when used in combination with traditional drugs [156], rather than as standalone treatments. In general, the efficacy of immunotherapy depends on the immunosuppressive environment developed by the tumor, the tumor’s sensitivity to the immunotherapy, and its capacity to trigger an immune response. The generation of immune responses against tumors involves targeting tumor neoantigens. Reversal of immunosuppression had a limited impact on the intratumoral decline in CD8^+^ and NK cell populations, with PD-1 expression on immune cell surfaces being a significant impact factor in this impact [157]. Key determinants for the efficacy of immunotherapy encompass the tumor’s composition, the localization of infiltrated immune components, and their respective functionality, all of which are essential for the precise targeting of the tumor immune system. In addition, the characteristics of the tumor stroma and blood vessels offer valuable insights into the strategic application of immunotherapeutics against the disease [12].

## 6. Characteristics of Immunotherapy

Immune cells from both the innate and adaptive immune systems infiltrate the TME and play a crucial role in maintaining the equilibrium of tumor dynamics, underscoring the importance of the immune system in immune surveillance. Immune responses can effectively eliminate threatening cells or diminish their morphologies and capacities [158]. However, cancer cells possess several sophisticated mechanisms, including immunosuppressive cell populations, upregulated negative regulatory pathways, and defects in the antigen presentation machinery [158], which enable them to evade immune surveillance by blocking the effector capacity of immune cells and abrogating antitumor immune responses. Immunotherapy, which aims to bolster natural defenses in eradicating malignant cells, represents a significant advancement in cancer treatment and has transformed the field of oncology. Although the concept of utilizing the host’s immune system to eliminate cancerous growths dates back a century [158], substantial progress has been achieved in contemporary scientific and clinical research. Immunotherapy has elicited clinical responses in various malignancies, albeit with low response rates and complex underlying mechanisms [159]. Currently, immunotherapy is a primary focus for certain cancers, as it functions by restoring the patient’s immune responses to combat tumor cells [160,161].

## 7. Mechanism of Immunotherapy for Pancreatic Cancer

Building upon a well-established immune system, various forms of immunotherapy have been developed over time. These include adoptive cell transfer, oncolytic viruses, immunostimulatory cytokines, and anti-tumor (bispecific) antibodies immunotherapies, such as mAbs. mAbs, which inhibit immunosuppressive signals from immune cells or cancer, are the most widely used in clinical practice today, with numerous FDA approvals for solid tumors. Their anti-tumor effects are further enhanced by ICI treatment [162,163]. These therapies have demonstrated stimulatory and durable effects in certain patients by modulating their immune response [164]. The mechanisms of immunotherapy developed thus far include blocking immune checkpoint proteins, enhancing the efficiency of antigen recognition by immune cells, increasing intra-tumoral T cells, and administering tumor-specific antigens through vaccines to aid the immune system in recognizing tumor cell types [130,165]. Specific active immunotherapies target the adaptive immune system by activating T or B cells against the respective antigens of the PC [166]. mAbs, immune checkpoint-blocking antibodies directed against a specific protein/peptide of the PC, function as specific passive immunotherapies. T cells can prime antigenic targets when administered to patients to elicit an immediate response, thereby circumventing the need for antigen presentation. Recently, mRNAs have also been used as new targets for passive immunotherapy. Nonspecific adoptive immunotherapies, as the term suggests, involve the adoptive transfer of specifically engineered immune cells or immune cells coupled with cytokines or lymphokines to a patient with PC [167]. Recent studies have discussed in detail the potential of targeting DCLK1 to inhibit cancer stem cell self-renewal in tumors [168,169].

### 7.1. T-Cell Mediated Therapy

Immunotherapy strategies for pancreatic adenocarcinomas, including the reactivation of NK cells, the promotion or reintroduction of DCs, vaccination techniques utilizing DCs, and the reconstruction of the TME with a focus on macrophages, as well as controllers mediated via cancer or stromal cells, have the potential to modify the cancer microenvironment and enhance T-cell immunity [170]. T-cell therapy employing chimeric antigen receptors (CARs) targeting CD19 and B-cell maturation antigen (BCMA) has significantly improved the treatment of hematologic malignancies. However, the transfer of CAR-T cells targeting overexpressed PC antigens such as mesothelin, CD133, and epidermal growth factor receptors (EGFRs) have proven ineffective in patients with PC [171]. The CAR modulator protein comprises an intracellular segment with specific signaling domains, such as CD28 and CD137 (4-1BB), for T-cell activation, and an extracellular portion that recognizes a cell surface protein [172]. It has been established that 72% of PDAC express CD24, a small, mucin-like, heavily glycosylated, glycosylphosphatidylinositol-anchored cell surface protein associated with higher tumor grades [173], along with CD44 and CD133 [174]. In a human PC xenograft model utilizing orthotopic transplantation, the application of CAR-T cells incorporating anti-CD24 single-chain Fv and CD28 extracellular domains, in conjunction with light exposure, IL-2 administration, and cancer elimination, has been demonstrated [170]. It has been shown that the combination of ICI and chemotherapy is more effective than either treatment alone. The administration of folfirinox and PD-L1 significantly increased overall survival rates and elevated the number of CD8^+^ T cells and PD-1 expression in tumor and immune cells [175]. Another study indicated that the combination of BL-8040 and pembrolizumab with chemotherapy resulted in a disease control rate of 77% [176].

A previous study has suggested that chemotherapy and radiation enhance the responsiveness of cancers to ICB therapy through the release of pathogen associated molecular patterns (PAMPs) and damage associated molecular patterns (DAMPs), which drive TLR-dependent [177] and stimulator of interferon gene (STING)-dependent DC activation. In particular, the activation of the cyclic GMP-AMP synthase (cGAS)-STING pathway by cytosolic tumor DNA is essential for the formation of antitumor T effector cells and the efficacy of ICB therapy in preclinical cancer models [178]. Current research is exploring preclinical strategies to activate the cGAS-STING-IFN axis as an adjunct to chemotherapy, radiation, and immunotherapy, despite the lack of clinical data [179]. By enhancing MHC-I expression, antigen cross-presentation, and T-cell priming, the tumoricidal effects of chemotherapy and radiation synergize with immunotherapy to augment T-cell receptor (TCR) diversity and improve T effector cell infiltration [180]. The dosage and sequencing of chemotherapy and radiation are likely to significantly impact the antitumor immune response. In preclinical models of ovarian cancer, metronomic dosing of chemotherapy is considered the optimal dosing strategy, as it maintains immune function and enhances the activity of T effector cells [181]. This approach is commonly employed to improve cancer vaccine responses by reducing the abundance and inhibitory capacity of Tregs. Furthermore, hypofractionated irradiation diminishes the induction of the DNA exonuclease Trex1, facilitating the cytoplasmic accumulation of double-stranded DNA, thereby-promoting STING-mediated type I IFN release by cancer cells, which is superior to single-dose treatment regimens in preclinical mouse models concerning the CTLA-4 blockade [182]. In addition, patients receiving a combination of Ipilimumab and GVAX/CRS-207exhibited an expanded T-cell receptor repertoire in their peripheral blood [183]. Tumor-infiltrating cells (TILs) proliferate following the administration of PD-1 blockade, 4-1BB stimulation, and CD8^+^ induction therapy, characterized by IFN-γ expression [107,184]. Enzymes that degrade amino acids, such as arginase and indolamine 2,3-dioxygenase (IDO), are targeted to disrupt the TME of PC due to their detrimental effects on the tumor. Adenosine-reducing agents (e.g., oleclumab) and antineoplastic drugs (e.g., urelumab, varlilumab, and zanolimumab) influence anti-tumor T-cell responses [185]. The increased density of cytotoxic T cells in both inflamed and non-inflamed tumors indicates that the immune checkpoint inhibition is a promising strategy for enhancing T-cell immunity [186,187].

### 7.2. Macrophage Programming

Human PDAC is characterized by extensive immune cell infiltration and fibrosis within its microenvironment of. Among the infiltrating cells, macrophages represent a significant component, and their inhibition may offer a promising strategy for reconstituting the cancer microenvironment [170]. In a mouse model of PC driven by mutant KRAS/p53 [doxycycline-inducible Kras G12D and Trp53 R172H under the control of pancreatic transcription factor 1 (Ptf1), Cre recruiter], the depletion of CD11b^+^ myeloid cells resulted in the expansion of intratumoral CD8^+^ T cells [188]. The metabolic reprogramming of macrophages can lead to the methylation of the *Nqo-1* gene, thereby initiating protumor activity in a pancreatic model [189]. Furthermore, CAFs frequently develop resistance to FLOFIRINOX treatment, and reprogrammed macrophages contribute to reduced PC survival [190].

### 7.3. Antibody and Immune Checkpoint Inhibitors

**Figure 3:**
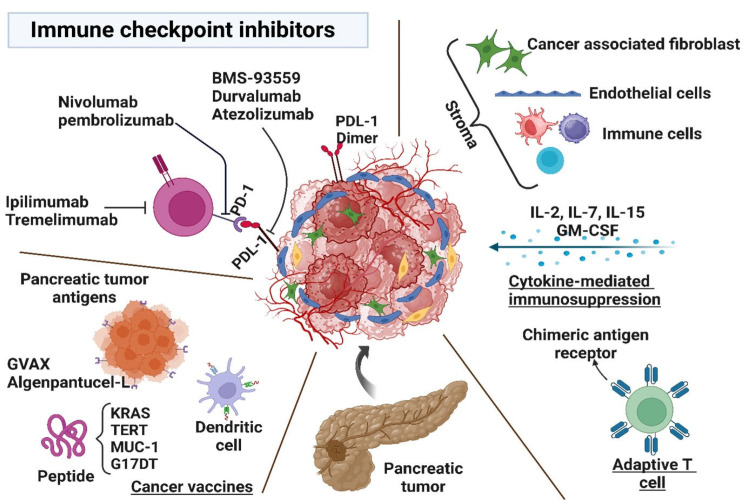
Immunotherapy for pancreatic tumors. Various therapeutic approaches for the treatment of PC are shown. The blockade of Immune checkpoints using anti-PD-1, anti-PD-L1, and/or anti-CTLA-4 agents facilitates the activation of T cells, representing a promising approach for the immunotherapy of pancreatic tumors. Ipilimumab and tremelimumab are fully humanized mAbs targeting anti-CTLA-4 IgG1 and IgG2, respectively, and are approved for clinical application. Therapeutic cancer vaccines, which present immunogenic cancer antigens to the immune system, can induce in vivo activation of cytotoxic T lymphocytes specific to cancer antigens, thereby eliciting an anti-cancer immune response. Granulocyte-macrophage colony-stimulating factor (GM-CSF) is expressed by pancreatic tumor cells engineered to produce GVAX, a whole tumor cell vaccine. PDAC is characterized by a dense stromal/desmoplastic response, involving a multitude of diverse cells, including fibroblasts, immune cells, PSCs, extracellular matrix, and numerous soluble proteins, including growth factors and cytokines. Stromal synthesis may account for 90% of the overall tumor volume. The most clinically advanced form of adoptive cell transfer (ACT) is chimeric antigen receptor (CAR) T-cell therapy, wherein the T cells of clinical participants are genetically modified to express CAR on their surface.

Innate anti-tumor immunity can be enhanced through the application of tumor-targeted mAbs. Tumor cells often evade immune surveillance by activating immune checkpoint pathways, which suppresses antitumor immune responses and allow for unchecked tumor progression. A notable advancement in cancer immunotherapy involves the use of FDA-approved mAbs that inhibit immunological checkpoints (ICIs), thereby augmenting antitumor immune responses to eliminate tumor cells [191,192]. mAbs utilized in PC immunotherapy include ipilimumab, nivolumab, tremelimumab, atezolizumab, and pembrolizumab, all of which function as ICIs [16,193]. In addition, the CD40 antibody, expressed in most hematopoietic and non-hematopoietic cells and tissues, is recognized for its role in regulating immunity [194]. A previous study has shown that CD40 signaling and activation lead to the generation of cytotoxic T lymphocytes [195]. Ipilimumab and tremelimumab specifically target Cytotoxic T-lymphocyte-associated protein 4 (CTLA-4). The efficacy of single-agent therapies is generally lower compared to combined checkpoint inhibitor therapies [196]. The interaction between PD-1 and CD80 is disrupted through ICP therapy. Mesothelin (MSLN) is a protein ubiquitously expressed various cancer types. MUC16 serves as the ligand of MSLN, and mAbs targeting MSLN, and mAbs targeting MSLN can inhibit the binding of MSLN to mucin 16 (MUC16), thereby impeding its function. This also facilitates antibody-dependent cytotoxicity (ADCC) [88,197] (Figure 3).

The modulation of ICI receptors and co-inhibitory molecules on T cells is crucial for maintaining immunological homeostasis and regulating the functions of effector and regulatory cells [198]. Achieving a balance between immune effector and immunosuppressive cells within the TME is essential for effective cancer immunity. The immune response is regulated by the interactions between immune cell receptors and costimulatory ligands. The CD28 family also includes the T cell Ig and immunoreceptor tyrosine-based inhibition motif (ITIM) domain (TIGIT), a co-inhibitory particle that interacts with T cells and NK cells [199]. Although extensive clinical trials have been conducted using ICIs to treat PC, their overall effectiveness has not been satisfactory [200,201]. The immunosuppressive microenvironment of PC contributes to the limited efficacy of ICIs targeting PD-1/PD-L1 [202]. The PC microenvironment is characterized by a dense interstitial matrix, which acts as a physical barrier to T and NK cells, enabling PC cells to evade host immune surveillance. This highly repressive microenvironment not only facilitates immune invasion by tumor cells but also hinders drug penetration and their anti-tumor efficacy. PC is inherently less immunogenic [203,204,205]. As a result, numerous studies have sought to enhance the immunogenicity of PC to improve the effectiveness of immunotherapy [206].

### 7.4. Vaccines

**Figure 4:**
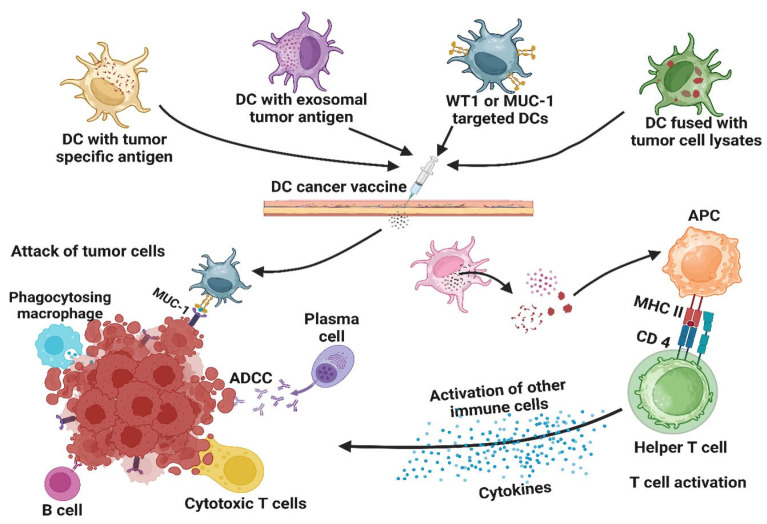
DCs as a cancer vaccine targeting PC cells. DCs can be loaded with tumor-specific antigens and exosomal antigens. In addition, the fusion of tumor cell lysates with DCs, as well as WT1- or MUC-1-targeted DCs, can be produced. Following the administration of the DC vaccine, the released tumor-specific antigens are presented in the bloodstream by APCs to helper T cells, thereby initiating cell-mediated immunity against pancreatic tumor cells. DCs directed against MUC-1 have the capability to bind directly to the MUC-1 receptors present on the tumor cells, thus eliciting an immune response.

A promising approach to hindering the progression of PC involves the development of cancer vaccines utilizing antigen-specific T cells, which are introduced into the TME [207]. T cells are capable of recognizing antigens when presented by APCs that degrade an endogenous or exogenous mutant peptide produced by the tumor, followed by its processing and presentation via the MHC complex on the cell. DCs serve as APCs involved in the processing and presentation of antigens to CD8^+^ T cells, which are recognized by the *αβ* T-cell receptor (TCR) [208]. The significance of DCs as a PC vaccination has been emphasized by numerous studies. The study by Jang et al. highlights the importance of DCs in PC regression [209], demonstrating that the suppression of Treg cells (which interact with and impair the function of DCs) enhances the anti-tumor activity of CD8^+^ T cells by restoring the immunogenic function of CD11^+^ DCs, thereby inhibiting tumor growth. Due to various limitations associated with the use of DCs as vaccines, mature DCs have been developed in vitro and employed as cancer vaccines because they can effectively produce tumor-specific antigens for recognition by CD4^+^ and CD8^+^ T cells. To enhance the specificity of DCs and their efficiency in antigen presentation, different cell components or whole cells have been fused with DCs. The fusion of DCs with tumor lysates has been shown to induce a generalized anti-tumoral T-cell response in patients with PC, thereby extending overall survival by 56 months in one patient and achieving a 1-year survival in 5 out of 12 patients [62]. DCs have also been loaded with tumor antigens, such as exosomes [84], and fused with cancer cells to form hybrids [85]. In addition, DCs have been utilized to target various components of the TME. Most clinical trials have been conducted with MUC1- and WT1-targeted DCs, primarily in phase I/II trials [210,211]. MUC1 was used either as a peptide or as cDNA with DCs. Either MHC I or MHC II or both classes, restricted WT1 in combination with DCs for therapies (Figure 4). The results indicated that patients were able to respond to treatment with minimal or very few side effects [86]. Based on several characteristics of WT1, such as its therapeutic efficacy, specificity, immunogenic activity, role in tumor progression, the presence of a high number of positive WT1 antigens, and expression of WT1 by cells, the National Cancer Institute (NCI) has ranked this antigen as the top antigen for use as a vaccine [87]. Another target, hTERT/DC mRNA, when administered for 3 years to patients who could not receive chemotherapy, resulted in no active disease and an effective immune response [91].

Carcinoembryonic antigen (CEA) is a glycosylated protein present in 90% of PCs and represents a potential therapeutic target [92]. KRAS mutations, which are expressed in over 99% of PCs, pose a significant challenge for therapeutic intervention [93]. Vaccination targeting with Kras has shown a 9-year memory response post-vaccination and a 10-year survival rate with an immune response following surgical resection [97]. Beyond antigenic components used in vaccines, whole pancreatic tumor cells are also employed to elicit an immune response against cancer. In a study involving 93 patients, GVAX was administered with cyclophosphamide in combination with CRS-207, with or without nivolumab. Although no significant results were observed, the outcomes were comparable to those of standard therapy. Notably, patients treated with arm A (Cy/GVAX^+^CRS-207^+^ nivolumab) exhibited a superior immune response, characterized by increased CD8^+^ T cells and decreased CD68^+^ myeloid cells [98].

GVAX, in conjunction with ipilimumab, facilitates the differentiation of T cells into effector variants and enhances the presence of M1 macrophages within the tumor, although the anticipated overall survival was not achieved [58]. Another investigation explored the combination of GVAX and cyclophosphamide with a chemotherapeutic agent selected by the physician in various combinations. No improvement in overall survival was observed, but side effects such as chills, pyrexia, fatigue, and nausea were reported [103]. An alternative approach to inducing an immune response involves chemotherapeutic agents that induce tumor cell death by releasing cellular components, thereby activating DCs and enhancing the processing and presentation of relevant tumor-associated antigens (TAA) [105]. Patients with advanced PC do not benefit from the telomerase peptide vaccine GV1001, the Granulocyte-Macrophage Colony-Stimulating Factor (GM-CSF)-transfected pancreatic tumor vaccine (GVAX), or an allogeneic whole-cell vaccine derived from a PDAC cell line genetically engineered to express GM-CSF [212]. Pre-vaccination before immune checkpoint therapy enhances the effect of the infiltrating immune checkpoints, thereby improving vaccine efficacy. In addition, immune checkpoint therapies have been combined with DC vaccines to augment the antigen-presenting efficiency of cytotoxic T cells. The limited number of neoantigens in tumors presents a challenge in the development of cancer vaccines [104]. Most cancer vaccines remain in clinical trials, as further understanding is required regarding the interplay between host and tumor immune systems, the TME, and administered therapeutics.

### 7.5. Cytokine Therapies

The TME can be profoundly affected by the targeted delivery of cytokines [213]. Due to their short half-life, cytokines function in an autocrine or paracrine manner, necessitating their concentration at specific sites. However, several cytokine-based therapies employed in clinical practice, such as IL-2 and IFN, are administered systemically, often resulting in serious dose-limiting toxicities [214]. The challenges in directing cytokines to the appropriate site and the incomplete understanding of the effects of specific cytokines across various cancer types constrain the effectiveness of cytokine-based therapies [215]. GM-CSF is instrumental in selecting and activating antigen-presenting cells that process and present cancer-associated antigens, thereby eliciting an effector T-cell response. Talimogene laherparepvec (T-VEC) is a modified herpes simplex virus type 1 (HSV-1) that encodes the gene for human GM-CSF [216]. The intratumoral administration of this engineered HSV prompts cancer cells to produce extended levels of GM-CSF locally, which attracts antigen-presenting cells through chemotaxis, initiating a local adaptive immune response to tumor antigens capable of cancer distant from the infusion site. In animal models, this approach confers protection against reinfection with the same tumor [217].

### 7.6. Novel Checkpoint Blockade Targets

T-cell immunoglobulin 3 (TIM3), lymphocyte enactment gene 3 (LAG3), B7-H3, the V-domain immunoglobulin suppressor of T-cell activation (VISTA), and T-cell immunoreceptors with immunoglobulin and immunoreceptor tyrosine-based inhibitory motif domains (TIGIT) are among the recently identified negative regulators of T-cell activation currently under investigation [218,219,220]. Two well-established mechanisms that mediate T-cell immune checkpoint suppression are the PD-1/PD-L1 and CTLA-4/B7 signaling pathways. Beyond these two pathways, the interaction between tumor cells and host immune cells involves numerous immunomodulatory receptor-ligand interactions, which can be targeted clinically through either monotherapy or combination therapy [221]. LAG3, an inhibitory ligand present on activated T cells and Treg cells, inhibits CD4 binding sites on MHC class II proteins, thereby attenuating T-cell activation. It induces cell cycle arrest, preventing excessive expansion of the T cell compartment [222]. TIM3 is another negative controller of T-cell responses, likely modulating apoptosis following galectin 9 binding by restricting cell cycle progression, akin to LAG3 [223]. The upregulation of TIM3 may contribute to resistance against anti-PD1 treatments, suggesting that combination therapy could enhance the efficacy of anti-PD1 treatments. Moreover, TIM3 expression is associated with poor prognosis in follicular lymphoma and non-small cell lung cancer, indicating its role in the cancer progression (201). B7-H3 is another target that negatively regulates T-cell responses and is highly expressed in various cancers, including non-small cell lung, pancreatic, prostate, colorectal, and ovarian cancers [219,220,224]. Due to the presence of two immunoreceptor tyrosine-based inhibitory motifs in its intracellular domain, TIGIT suppresses T-cell hyperactivation and is being explored as a potential checkpoint target. TIGIT is more strongly expressed in TILs than in peripheral cells, making it a promising target due to its higher specificity compared to other checkpoint molecules [220].

## 8. Suppression of Immunity

**Figure 5:**
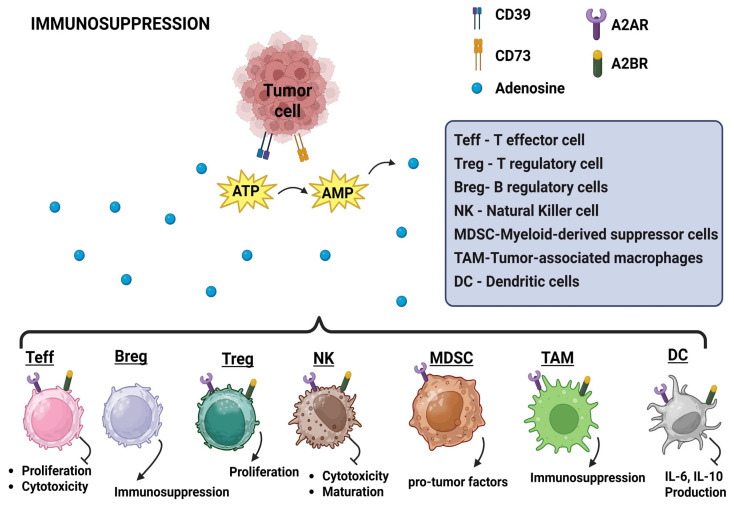
Immunosuppression. CD73 is expressed in various cell types within the TME and serves as a significant immunosuppressive agent by generating extracellular adenosine. Adenosine, through its interaction with A2AR and A2BR receptors on diverse immune cells, impairs the function of immune cells that defend against tumors (such as effector T cells, NK cells, DCs, and B cells), while enhancing the activity of immune cells that suppress the immune response (such as Treg cells, TAMs, MDSCs, and CAFs).

Through various mechanisms, cancer cells cause both local and systemic immunosuppression, which can significantly impact the efficacy of immunotherapy in clinical settings. The production of surface particles such as PD-L1, which leads to the exhaustion of tumor-infiltrating CD8^+^ T lymphocytes and NK cells, is a particularly critical factor [225]. In certain instances, such as patients with non-small lung cancer (NSCLC) undergoing treatment with ICBs, the expression of PD-L1 in malignant cells serves as a potent prognostic marker [226]. The plasma membrane protein 5′-nucleotidase ecto (NT5E; also known as CD73) plays a role in the conversion of extracellular ATP—an agent with strong chemotactic properties that targets DCs—to adenosine, contributing to the accumulation of malignant cells in an immunosuppressive microenvironment. CD73 also modulates the balance between immunostimulatory adrenergic signaling and its immunosuppressive adenosinergic counterpart by inhibiting resistance responses through various mechanisms [226,227]. When adenosine activates the A2A adenosine receptor (A2AR) receptor on effector T cells, it suppresses their activation and proliferation, leading to T-cell anergy [228,229]. In addition, A2AR activation impairs the maturation, activation, and cytotoxic functions of NK cells [230,231]. Additionally, naive CD4^+^ T cells activated by A2AR are more likely to differentiate into regulatory T cells (Treg) expressing the markers Foxp3+ and LAG-3+ [232,233]. Furthermore, through the A2BR receptor, adenosine produced by CD73 enhances the immunosuppressive effect and promotes the infiltration of MDSCs in tumors [234,235]. Regulatory B cells (B reg) are integral to the suppression of immune responses across a range of diseases [236,237]. Targeting B cells may reduce the burden of PC [238]. Induced B reg cells have natural killer function against tumor cells [239,240]. Recently CD38^+^ B cells reduced the antitumor immunoreactivity in PC [241]. Overall, CD73 attenuates the immune response to tumors by diminishing the cytotoxic activity of effector cells and enhancing the function of regulatory immune cells [234]. Consequently, CD73 represents a promising target for cancer immunotherapy (Figure 5). Various immunosuppressive agents in TME and their targets cells mentioned in Table 2.

## 9. Aptamer-Based Immunotherapy

To expand the applicability of immunotherapy in oncology, preclinical studies consistently indicate that multidimensional strategies employing a combination of various immunomodulatory agents should be considered. The potential for severe autoimmune-like reactions inevitably increases with the administration of multiple immunostimulatory drugs. Consequently, the development of target-specific therapeutic molecules with fewer adverse effects than mAbs is imperative. Oligonucleotide aptamers may address this limitation, as suggested by current preclinical research [261]. Aptamers are single-stranded DNA or RNA oligonucleotides with three-dimensional structures that exhibit high affinity for their targets. They are selected through a process known as Systematic Evolution of Ligands by Exponential Enrichment (SELEX) [262], an exceptional filtration technique that isolates rare particles from a vast, complex library [262]. The concept of aptamer-conjugated oligonucleotide backbone can be applied using DNA and/or RNA. To enhance the stability of aptamers in serum, several modifications can be implemented, such as substituting 20-amino, 20-fluoro, or 20-O-alkyl nucleotides for ribonucleotides. Oligonucleotide-mediated aptamers offer distinct advantages over mAbs alone. As cell-based products, mAbs are associated with higher production costs and a complex administrative burden to achieve good manufacturing practice (GMP), whereas aptamers, being synthetically derived molecules, resulting in lower production costs. Consequently, they offer the potential for less burdensome administrative approvals and most cost-effective alternative reagents for future clinical trials [261]. Aptamers have been employed as ligands in non-invasive diagnostic tests, such as ELISA and other immunoassays commonly used to analyze biomarkers in blood samples. These novel “aptasensors” or aptamer-based assays can be equipped with readout techniques, such as chemiluminescence (CL), electrochemiluminescence (ECL), fluorescence, surface plasmon resonance (SPR), surface-enhanced Raman spectroscopy (SERS), among others, to enhance the detection of existing biomarkers [263,264,265,266]. Numerous innovative therapeutic approaches have been developed to optimize immunotoxicity and can be intensified in combination with immunotherapy. To address this challenge, one strategy involves the targeted delivery of immunotherapy to tumor or immune cells. In this context, aptamer–drug conjugates (ApDCs) are at a promising stage [267]. ApDCs have been utilized to deliver immunomodulatory agents to restrict immune system co-stimulation in the tumor region, inhibit neoantigens in cancer, block exhaustion-inducing immune checkpoints to revive functional immune cells and trigger anti-tumor immunity [267]. The identification of effective molecular targets, such as immune checkpoints and those that stimulate the immune system, presents the opportunity to enhance immunotherapy by focusing on specific molecular sites. Several aptamers have been developed for immunological checkpoints associated with immunosuppression in cancer patients, including T-cell immunoglobulin-3 (TIM-3), cytotoxic T lymphocyte-associated protein 4 (CTLA4), and programmed death receptor I (PD-1) or its ligand, programmed death ligand I (PD-L1) [268,269,270].

## 10. Immunogenicity

PC is characterized by low immunogenicity, indicating that it is not readily recognized or targeted by the immune system. Several factors contribute to this low immunogenicity, including TME, immune checkpoint signaling, tumor heterogeneity, stromal barrier, and antigen presentation [261,262,267]. Cancer neoantigens, which arise from somatic nonsynonymous DNA alterations unique to cancer, are often the focus of effective adaptive immune responses against cancer cells [271]. Consequently, an increased mutational load, which enhances the likelihood of neoantigen emergence, has been correlated with heightened sensitivity to ICB-based immunotherapy in various clinical studies [225,272]. In addition, immunogenic chemotherapy and radiotherapy elicit multifaceted immune responses partially through pathogen mimicry. In this context, cancer cells undergoing treatment-induced stress responses release several endogenous molecules that function as adjuvants, including adenosine 5′-triphosphate (ATP), a downstream product of the autophagy process; calreticulin (CALR), a downstream product of endoplasmic reticulum stress; and type I interferon (IFN), a downstream product of Toll-like receptor 3 (TLR3) or cyclic GMP-AMP synthase signaling [273]. In addition, two considerations should be approached with caution as predictive biomarkers for cancer immunotherapy: (i) acute or chronic activation of certain signaling pathways may differentially influence immune responses in cancer, and (ii) the effects of a specific interaction on various types of immunotherapies may exhibit significant heterogeneity [274].

## 11. Factors That Limit the Efficacy of Immunotherapy

The effectiveness of immunotherapy in PC is limited by the relatively low number of aggregated mutations capable of inducing the expression of non-self-antigens, or “neoantigens,” which are recognized as foreign by the immune system. In comparison to malignancies with lower mutational loads, those with higher mutational burdens present a greater number of neoantigens, facilitating detection by the immune system [275,276]. There are three major limitations to immunotherapy for PC. Firstly, unlike lung cancer and melanoma, PC exhibits a very low mutational burden. Secondly, the immunosuppressive microenvironment, characterized by a dense desmoplastic reaction and a significant influx of tumorigenic macrophages and MDSCs, plays a significant role in the progression of pancreatic malignancy. Thirdly, the PC microenvironment is characterized by a paucity of infiltrating T cells, which hinders the development of adequate T-cell responses [277]. In addition to these factors limiting the efficacy of immunotherapy, other barriers to therapeutic success include the chronic inflammation associated with PC and the T-cell response to administered therapy [278]. When considering treatment with ICP blockade, only 4% of all tumor cells, including PC cells, CAFs, and CD163^+^ TAMs, express the PD-1 immune checkpoint [203].

In a groundbreaking study aimed at enhancing the efficacy of tumor immunotherapy, researchers focused on inhibiting the interferon gamma (IFN-γ) pathway in tumor cells. This suppression was found to enhance the overall effectiveness of immunotherapy and the body’s ability to identify and eliminate tumor cells through innate immune systems [203]. A recent study in the United States has made significant progress in understanding the role of IFNs in tumor immune responses. These findings suggest that IFNs influence the immune response to malignancies in two ways. Firstly, IFNs activate DCs, which are essential for delivering tumor-specific antigens to CD8^+^ T lymphocytes, thereby promoting cross-activation. However, prolonged exposure to IFNs can result in a negative feedback loop, leading to T-cell dysfunction and immunosuppression [278].

## 12. Future Perspectives

Although it is usually challenging to translate findings from preclinical models into clinical practice, the scarcity of new therapeutics being incorporated into the standard treatment for PDAC may particularly underscore the shortcomings of existing preclinical PDAC models. There are more ambitious translational research programs that run alongside clinical trials and enhance the understanding of PDAC immunology, even when clinical endpoints are not achieved. These programmes should ideally lead to “reverse translation” by experimentally establishing concepts that help overcome resistance to immunotherapeutic strategies. However, regarding translational methodology, it becomes evident that there are no established standards for sampling patient material and for evaluating immune responses. In addition, patient cohorts who have undergone novel treatment strategies have frequently received at least one previous line of therapy, involving repeated drug exposure. This feature, which can greatly influence the reaction to experimental treatments, is not replicated by most tumour models. Patient-derived organoid cultures or 3D cultures testing of the drugs/immunotherapeutic molecules may provide the better treatment options in patients with high degree of drug/immune resistance.

## 13. Conclusions

Recent advances in cancer immunotherapies, which stimulate the immune system to recognize and combat malignant tumors, have expanded the potential for effective treatment of such tumors. In recent years, the range of successful immunotherapies has increased, thereby broadening the therapeutic options available to oncologists. Despite significant progress in malignancy and translational research, PC continues to yield poor outcomes and retains highly lethal malignancy. The efficacy of immunotherapy in PC is currently under investigation in several early-phase clinical trials. These trials are evaluating the impact of adoptive cell transfer, ICIs, and their combinations with chemoradiotherapy or other molecularly targeted treatments. In many of these studies, cancer vaccines have consistently shown non-progressive outcomes. Future research should prioritize strategies to overcome immunotherapy resistance by addressing various immunodeficiencies and employing combined immunotherapy and cytotoxic approaches in patients with PC. At present, our understanding of the etiology of PC is limited, necessitating further comprehensive research. Large-scale studies are essential to elucidate the risk factors associated with PC. This study aims to identify novel diagnostic and therapeutic strategies that may enhance the survival of patients with PC.

## Figures and Tables

**Table 1:** Immunotherapies for pancreatic cancer clinical trials.

Phase	NCT	Settings	Interventions	Outcomes	Reference
II	NCT03190265	Second-line or later	Nivolumab +GVAX + CRS-207 + Ipilimumab + Cyclophosphamide vs. Nivolumab + CRS-207 + Ipilimumab	The study did not meet its primary endpoint of improvement in overall survival.	[98]
I/II	NCT02305186	Neoadjuvant	Chemoradiotherapy (with Capecitabine)	Adding pembrolizumab to neoadjuvant chemoradiotherapy was safe. However, no convincing effect on CD8^+^ TILs was observed.	[140]
II	NCT03727880	Neoadjuvant/Adjuvant	Pembrolizumab + Defactinib vs. Pembrolizumab	Pembrolizumab combined with defactinib was associated with lower fibroblast infiltration, higher anti-tumor M1 macrophage expression and increased CD8^+^ T-cell infiltration.	[141]
II	NCT03161379	Neoadjuvant	Nivolumab + Cyclophosphamide + GVAX + SBRT	Observed median OS, pCR and R0 resection rates were comparable to contemporary studies administering neoadjuvant mFOLFIRINOX and SBRT.	[142]
II	NCT03563248	Neoadjuvant	FOLFIRINOX → SBRT → Surgery vs. FOLFIRINOX + Losartan → SBRT + Losartan → Surgery	Downstaging of locally advanced pancreatic ductal adenocarcinoma.	[143]
II	NCT04940286	Neoadjuvant	Oleclumab + Durvalumab + Gemcitabina + Nab-Paclitaxel	Standard neoadjuvant therapy has the potential to improve outcomes in PDAC.	[144]
II	NCT05093231	First line	Pembrolizumab + Olaparib	PARP-inhibitor combinations, vaccines, and CAR-T-cells therapy provide some encouraging results.	[145]
II	NCT02648282	First line	Pembrolizumab + Cyclophosphamide + GVAX + SBRT	Phase II study of 54 pts w LAPC treated w CY/GVAX/pembro and SBRT. Primary endpoint of DMFS > 13.6 mos not reached, however 44% of pts underwent surgical resection of whom 42% had grade 1 path response rate.	[146]
IIb	NCT02907099	First line	Pembrolizumab + CXCR4 antagonist BL-8040	The increased T cell infiltration, CXCR4 antagonism was in fact associated with enrichment of CD206^hi^IA/IE^lo^ macrophage subtypes and modestly dampened efficacy	[147]
III	NCT03977272	First line	Anti-PD-1 antibody 200 mg + mFOLFIRINOX vs. mFOLFIRINOX	Sintilimab to mFFX improved ORR in advanced PDAC patients significantly, however no superior OS and PFS were observed.	[148]
II	NCT04377048	First line	Nivolumab + Gemcitabine + Tegafur-Gimeracil-Oteracil	Adding-on nivolumab was associated with improved OS in patients with advanced PDAC.	[149]
II	NCT04543071	First line	Cemiplimab, Motixafortide, Gemcitabine, Nab-Paclitaxel	Preliminary results from this pilot study of MCGN in mPDAC were promising, with a durable PR rate of 55% and disease control rate (DCR) of 82%, compared to historic PRs and DCRs of 23% and 48% reported with gemcitabine and nab-paclitaxel (GN), respectively.	[150]
II	NCT04177810	First line	Cemiplimab + Plerixafor	Mobilization of myeloid cells by CXCR4 antagonism results in the recruitment of additional myeloid cells from circulation and that alternative chemokine signaling pathways.	[151]
II	NCT04493060	First line	Dostarlimab + Niraparib	PARPi plus anti-PD1 checkpoint inhibition was not sufficiently active as later line therapy in metastatic pancreatic cancer for the majority of patients with HRD mutations.	[152]
I/II	NCT04827953	First line	Zalifrelimab + Gemcitabine + Nab-Paclitaxe + NLM-001	Reduced hypoxia and cancer cell contents in all pt and reduction in CAF, Tregs and macrophages in one pt.	[153]
II	NCT05014776	Second-line or later	Pembrolizumab + Ipilimumab + Tadalafil + CRS-207	PDE5 inhibition combined with vaccine-based immunotherapy promotes pro-inflammatory states of myeloid cells, activation of T cells, and enhanced myeloid/T cell crosstalk to yield antitumor efficacy against immune-resistant PDAC.	[154]
I/II	NCT04247165	Locally advanced	Nivolumab + Gemcitabine + SBRT + Nab-Paclitaxel + Ipilimumab	The combination was associated with good local control, low adverse event rate, and good QoL.	[155]

**Table 2:** TME and immunosuppression of pancreatic cancer.

Target Cells	Recruitment & Activation	Stromal Cells	Cell Derived Components	Immune Responses	References
Tregs, CD8^+^ T, TAMs, NK cells, tumor cells	Differentiated from fibroblasts, MSCs, adipocytes and PSCs	CAFs	CCL, IL-6, TGF-β, CXCL, MCP-1, IDO, PGE2.	Recruitment of Tregs and MDSCs, suppression of T cell and NK cell activity, along with heightened PD-L1 expression.	[242,243,244]
NK cells, CD8^+^ T, CD4^+^, and Tregs	VEGF, CXCL, CSF, IL, TGF-β, TNF-α, IFN-γ and PEG-2.	MDSCs	Arginase, iNOS, TGF-β, ROS, IDO, COX2, IL-6 and IL-10	Inhibition of lymphocyte function, recruitment of Tregs, and expression of immunosuppressive checkpoint molecules.	[245,246,247,248]
Tumor cells, CD8^+^ T, Tregs	CXCL, CCL, TLR4, VEGF, IL-4, IL-13	TAMs	TNF I, FN-γ, iNOS, MHCII, ARG1, IL-10, PGE2, EGF, EGFR, TGF-β IL-10, CD163 and CD204	Causing Treg differentiation, preventing T cell activity, generating inhibitory cytokines, and raising the expression of CTLA-4 and PD-1.	[249,250,251]
CD8^+^ T, Th cells, NK cells, and APCs	CXCR3, CCL9/10/11, CXCL10, CCR4-CCL17/22 and CCR8-CCL1	Treg cells	IL-10, PD-L1, TCF-β, CTLA4, MADCAM-1, VCAM-1, granzyme B and perforin	Immunosuppressive cytokine secretion, NK cell apoptosis induction and immune cell function inhibition.	[252,253]
Tregs, CAFs, and TAMs	CXCL, TGF-β, IFN-β and GM-CSF	TANs	IL-13, CCL17, CCL2, ARG1, elastase and MMP9	Advancing the polarization of TAMs, Treg recruitment, and the up-regulation of ARG1 and PD-1.	[7,254]
Immune cells, tumor cells, TAMs, MDSCs	interleukin and TGF-β,	PSCs	IL-10, CXCL12, MCP-1, VEGF, fibronectin and type I collagen	Encouraging the differentiation and migration of MDSCs and TAMs, resulting in an imbalance of Th1/Th2 cytokines	[255,256]
T cells, Tregs, Th17.	Recruitment is inhibited by PGE2	DCs	CD80, CD40, CD70, CD86, MHC-I, MHC-II, IFN-γ, IL-12, IL-15, MGL2 and PD-L2.	Impairment of DC activation, maturation and the display of antigens; Encouraging the multiplication of Treg cells while hindering immunity mediated by CD8^+^ T cells; controlling the equilibrium between Th17 and Treg cells.	[257,258]
T cells, DCs, macrophages.	CD47, HLA-G, CCL27/CCR10, CCL5/CCR5, CX3CL1/CX3CR1, ECM	NK cells	GM-CSF, IFN-γ, TNF-α, IL-3, perforin and granzyme	MDSCs and Treg cells prevent NK cell toxicity through TGF-β and inhibitory signals.	[259,260]

## Data Availability

The original contributions present in this study are included within the article. Further inquiries can be directed to the corresponding authors.
